# Why Use Adipose-Derived Mesenchymal Stem Cells in Tendinopathic Patients: A Systematic Review

**DOI:** 10.3390/pharmaceutics14061151

**Published:** 2022-05-27

**Authors:** Annalisa Itro, Maria Consiglia Trotta, Roberta Miranda, Marco Paoletta, Annalisa De Cicco, Caterina Claudia Lepre, Umberto Tarantino, Michele D’Amico, Giuseppe Toro, Alfredo Schiavone Panni

**Affiliations:** 1 PhD Course in Translational Medicine (XXXV Cycle), Department of Experimental Medicine, University of Campania “Luigi Vanvitelli”, 80138 Naples, Italy; annalisa.itro@unicampania.it; 2 Department of Experimental Medicine, University of Campania “Luigi Vanvitelli”, 80138 Naples, Italy; caterinac.lepre@gmail.com (C.C.L.); michele.damico@unicampania.it (M.D.); 3 Department of Medical and Surgical Specialties and Dentistry, University of Campania “Luigi Vanvitelli”, 80138 Naples, Italy; roberta.miranda1@studenti.unicampania.it (R.M.); marco.paoletta@unicampania.it (M.P.); annalisadecicco24@gmail.com (A.D.C.); giuseppe.toro@unicampania.it (G.T.); alfredo.schiavonepanni@unicampania.it (A.S.P.); 4 Department of Clinical Sciences and Translational Medicine, University of Rome Tor Vergata, 00133 Rome, Italy; umberto.tarantino@uniroma2.it

**Keywords:** tendinopathies, tendons repair, adipose-derived mesenchymal stem cells, humans

## Abstract

The aim of the present systematic review was to provide a clear overview of the clinical current research progress in the use of adipose-derived mesenchymal stem cells (ASCs) as an effective therapeutic option for the management of tendinopathies, pathologies clinically characterized by persistent mechanical pain and structural alteration of the tendons. The review was carried out using three databases (Scopus, ISI Web of Science and PubMed) and analyzed records from 2013 to 2021. Only English-language papers describing the isolation and manipulation of adipose tissue as source of ASCs and presenting ASCs as treatment for clinical tendinopathies were included. Overall, seven clinical studies met the inclusion criteria and met the minimum quality inclusion threshold. Data extraction and quality assessment were performed by groups of three reviewers. The available evidence showed the efficacy and safety of ASCs treatment for tendinopathies, although it lacked a clear description of the biomolecular mechanisms underlying the beneficial properties of ASCs.

## 1. Introduction

Tendinopathies is an umbrella term that includes several different clinical entities characterized by persistent mechanical pain and structural alteration of the tendons [1,2], a tough band of bright white fibro-elastic tissue connecting the muscle to the bone [3].

Tendon disorders cause an invalidating musculoskeletal pain that represents a common reason for physician consultation [4]. They severely impact the patient’s daily life, thus being associated with an increase in both direct and indirect social costs [5,6,7]. However, the prevalence of tendinopathies is probably underestimated, considering the increase in sport activities. It represents approximately 30–50% of sports injuries [4].

The pathogenesis of tendinopathies is clearly multifactorial [6]. A genetic component was reported in Achilles, elbow and rotator cuff tendinopathies [8,9], although genetic modifications alone are not able to justify the insurgence of the disease [8]. Indeed, the possibility of developing a tendinopathy seems to be increased by the interaction between intrinsic (age, body structure, nutrition, metabolic, genetic) and extrinsic factors (fatigue, improper loading, disuse, exogenous damage) [9,10]. Among them, tendon overload in sports or in specific work activities has been reported to be a crucial factor promoting tendinopathies [11,12]. Similarly, some metabolic and hormonal imbalances, including diabetes, thyroid hormones, estrogens, testosterone, growth hormone, cortisol, cholesterol and glutamatergic changes, occur [13,14,15,16,17,18,19]. Inflammation also seems to play a critical role in tendon degeneration [6]. In fact, although a presence of inflammatory cells in injured tendons has not been found [4], it is likely that changes in the balance between prostaglandin E (associated with tendon inflammation and pain) and prostacyclin occur [20]. However, the exact mechanisms leading to tendinopathy are still unclear.

Clinically, tendinopathies are characterized by activity-related pain, localized tenderness, swelling and reduced performance [21]. However, it is not uncommon to find completely asymptomatic patients with complete tendon degeneration [6,7].

The management of tendinopathies is challenging and often frustrating. Conservative treatment, based on reduced activity, is generally preferred at the beginning of symptoms [22,23] and is frequently associated with non-steroidal anti-inflammatory drugs (NSAIDs) [24]. However, there is no clear evidence on the long-term benefits for tendinopathies for both NSAIDs and steroids, with corticosteroid injections being useful, especially for short-term pain relief [24,25]. In the long term, however, they could even be responsible for tendon rupture [26]. Interestingly, although not considered in the current recommendations for tendinopathies management, hyaluronic acid (HA) has emerged as a novel therapeutic option [27]. Indeed, several clinical studies reported promising results obtained with HA injections in patients with rotator cuff, supraspinatus and Achilles tendinopathies, lower limb tendon injuries and lateral epicondylitis, as well as in sport-related ones [27]. The rationale for HA use in tendon injuries is related to its analgesic, repairing, anti-inflammatory and proliferative effects [28], along with a good safety and tolerability profile [27,28,29,30,31].

Although several options are available for the management of tendinopathies, both conservative and surgical, their treatment is still a challenge for the muscle skeletal physician [5,32]. A potential turning point might be represented by the use of adipose-derived mesenchymal stem cells (ASCs) [33]. Being easy to harvest and transplant, the ASCs are widely used for several clinical applications, including regenerative medicine [33,34,35,36]. Specifically, ASCs can be easily isolated from abdominal subcutaneous adipose tissue by lipoaspiration; the tissue harvested is then washed, filtered through a specific system and then applied where needed (i.e., knee, hip, first metatarsal joint) [35,36,37,38,39,40,41]. Under specific conditions, ASCs can differentiate into different cell lines, such as adipocytes, osteoblasts, chondroblasts, hepatocytes and neuronal cells [42]. In addition, ASCs seem to be able to differentiate into tenocytes and to enhance tendon repair [43]. Although the molecular mechanisms related to the tenogenic differentiation of ASCs are poorly understood, their stimulation by some specific growth factors, such as, for example, the connective tissue growth factor (CTGF) and tenascin-C (TN-C), seems to act by promoting the ASCs differentiation into tendon fibroblasts and improving tendon regeneration [43,44,45]. Moreover, the stimulation of ASCs cells determines the activation of specific molecular pathways that regulate the dynamic reorganization of the actin in the cytoskeleton of the tenocytes, promoting their migration [46,47].

Therefore, the use of ASCs may be extremely useful in tendon regeneration and repair [42]. To date, however, the evidence supporting the use of ASCs in tendinopathies is still scarce and mostly based on in vivo and in vitro studies [45,48,49,50,51,52]. Therefore, the aim of the present systematic review was to summarize the available clinical evidence that proposes and evaluates the application of ASCs in tendinopathies in order to give a clear overview of the present research and progress. This was performed by analyzing the type of adipose tissue used as source of ASCs, the type of ASCs injections in tendinopathic patients, the score evaluating tendinopathy at follow-up and the adverse reactions after ASCs treatment.

## 2. Methods

This review followed the PRISMA guidelines [53], and the protocol developed was published in the PROSPERO database (ID: CRD42022333131). The correspondent checklist is presented in the Supplementary Material (Table S1).

### 2.1. Eligibility Criteria

The inclusion criteria for the studies were: (1) English as language of publication; (2) tendinopathy as target disease; (3) clinical studies involving patients with tendinopathies; (4) evaluation of tendinopathy with magnetic resonance imaging (MRI), ultrasonography (US) and/or clinical scores; (5) description of autologous or allogeneic adipose tissue isolation and manipulation for ASCs isolation; (6) description of ASCs isolation or characterization in other cell fractions from adipose tissue; (7) evaluation of ASCs as treatment for tendinopathy; (8) long-term follow-up (at least 6 months). The authors only considered research articles, excluding editorials, book chapters and reviews. The primary outcome considered was the measure of ASCs therapeutic efficiency in tendinopathy, while the secondary outcome was related to the adverse events of ASCs treatment.

### 2.2. Information Sources and Search Strategy

The literature search was carried out by using 3 electronic databases as information sources (ISI Web of Science, Scopus and PubMed) and was based on a pre-determined series of keywords related to tendinopathies and ASCs application. The keywords used in PubMed were: “tendon” OR “tendon pain” OR “tendinopathy” OR “tendinopathies” AND “Adipose-derived mesenchymal stem cells”, by adapting them to subject headings or syntax of Scopus and ISI Web of Science. The literature search had no language or study design limits. It considered the period between 2013 and 2021. The date of last search was 29 December 2021.

### 2.3. Study Selection and Data Collection Process

During the selection of the studies, the search results were uploaded to Zotero, excluding duplicates. The results were then shared in a OneDrive folder, available to all the reviewers. The selection was composed of four stages. During the first stage, the eligibility criteria were applied to title, abstract and keywords, not excluding the studies of uncertain eligibility. During the second stage, eligible and uncertain studies were analyzed and screened in their full text, according to the inclusion criteria previously described. During the third stage, reviewers were randomized in groups of 3 in order to identify the suitable studies by consensus between at least 2 out of 3 researchers. Data extraction was then performed in the last phase. This was performed by discussing the relevant findings in a meeting with the whole research group. In case of any disagreement between the reviewers, findings were accepted when a consensus was expressed by all the reviewers for at least 8 of the 11 data items subsequently described.

### 2.4. Data Items and Quality Assessment

From each study included, the following data were extracted: (1) the type of clinical study; (2) the type of tendinopathy and its diagnosis; (3) the type of adipose tissue and its manipulation for ASCs isolation; (4) the type of cell fraction isolated from adipose tissue; (5) the ASCs treatment for tendinopathy; (6) the final follow-up; (7) the primary (scores evaluating tendinopathy) and secondary outcomes (adverse reactions reported) for ASCs treatment; (8) the number of treated and control patients at baseline and at final follow-up; (9) the characteristic of the patients (at least age and sex; body mass index—BMI—if reported); (10) the inclusion and the exclusion criteria; (11) the significant changes in scores at final and intermediate follow-up, confirmed by MRI or US.

Following the full-text selection, each of the 3 reviewers constituting a group independently assessed the methodological quality by using the criteria from the QualSyst tool for quantitative/qualitative studies [54] adapted to a systematic review (Table S2).

## 3. Results

Seven clinical studies were included in the current systematic review.

**Figure 1:**
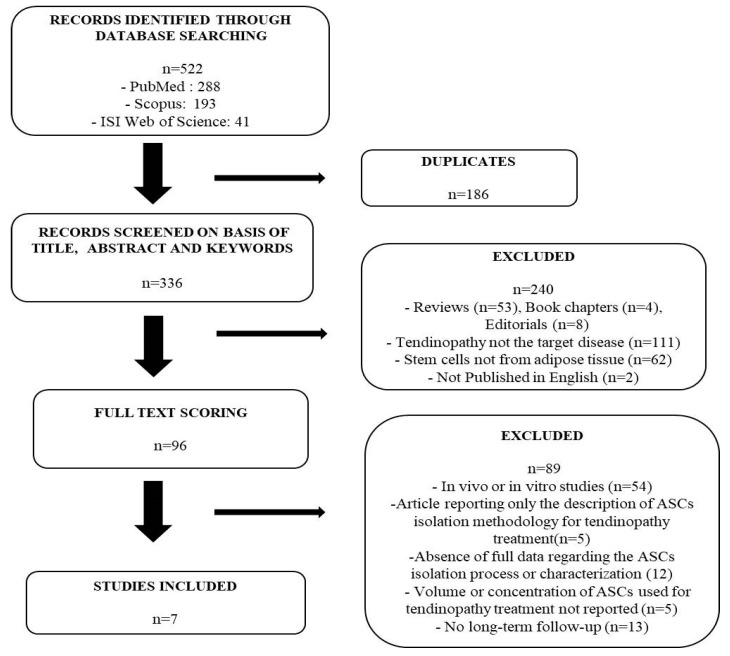
Flowchart diagram of the study selection process for this systematic review.

The search performed in the three databases (PubMed, Scopus, Isi Web of Science) retrieved a total of 522 records. They became 366 after 186 duplicate eliminations. Of these, 240 articles were rejected, since they did not meet the eligibility criteria during the evaluation of their title, abstract and keywords. Specifically, two studies were not included, since they were not published in English. The other rejected records included: 53 reviews and four book chapters, not considered in order to avoid bias derived from the analysis of the original published forms; eight editorials, which did not specify all the data related to the outcomes selected for the present review; 111 studies for which tendinopathy was not the target disease and 62 studies analyzing non-adipose-tissue-derived stem cells. Then, the remaining 96 full-text records were carefully evaluated with a screening and eligibility process. This rejected 89 studies. Of these 89, 54 described in vitro or in vivo experiences; 5 reported only a qualitative description of ASCs isolation methodology for their use in tendinopathy; 12 did not report full data about ASCs isolation process or characterization; 5 did not provide a complete description of ASCs volume or concentration; 13 did not consider a follow-up of at least 6 months. The resulting seven studies met the inclusion criteria and were considered eligible to be included in the present review (Figure 1). All the studies included met the minimum quality inclusion threshold (65%, see Table S2).

### 3.1. Study Design and Methods

The design and methods of the seven studies included in the present review are summarized in Table 1.

Among the seven studies included, three were performed in Korea [55,56,57], one in Italy [58], two in Argentina [59,60] and one in Australia [61]. In particular, two were classified as prospective longitudinal-case serious exploratory studies [59,60], one as cohort study [55], one as first-in human trial [56], one as randomized controlled clinical trial [58], one as open-label study [57] and one as case report [61]. The diagnosis of tendinopathy was performed in all studies by using MRI [55,56,58,59,60] or US [57,58,61]; three studies diagnosed elbow tendinopathy [57,60,61], two rotator cuff disease [55,56], one tendinopathy of Achilles tendon [58] and one patellar tendinopathy [59]. In the majority of the studies (six out of seven), the ASCs used for tendinopathy treatment were isolated from the autologous adipose tissue lipoaspirated from patients suffering with tendinopathy [55,56,58,59,60,61]. In two of these studies, it was specified that adipose tissue was harvested from the periumbilical zone [59,60]. Once lipoaspirated, autologous adipose tissue was processed by enzymatic digestion in five studies [55,56,59,60,61] and with a commercially available kit in one case [58]. In the remaining study, allogeneic subcutaneous adipose tissue was enzymatically digested after lipoaspiration [57]. Autologous or allogeneic ASCs (allo-ASCs) were isolated as cell fraction from adipose tissue in six studies [55,56,57,59,60,61] and used only after in vitro amplification in two of them [59,60]. In the remaining record, ASCs were not isolated as cell fraction but were characterized in stromal vascular fraction (SVF) isolated from adipose tissue [58]. All studies used intratendinous ASCs injection by choosing a volume of ASCs solution ranging from 1 to 4 mL [56,57,58,61]. This contained a cell concentration ranging from 1 × 10^6^ to 1 × 10^9^ ASCs [55,56,57,58,59,60,61]. Patients were evaluated after a final follow-up of 6 months in two studies [56,58], while in the other five studies, the final follow-up was increased up to 12 months [59,60], 13 [58], 28 [55] and 30 months [61]. The most frequent score used to evaluate the efficacy of ASCs treatment in tendinopathy, as primary outcome, was the visual analog scale (VAS) in all the clinical studies [55,56,57,58,59,60] except for the case report [61]. This was followed by the Victorian Institute of Sports Assessment questionnaire (VISA) and used in two studies [58,59].

**Table 1:** Study design and methodology of the included studies.

Reference	Study	Diagnosis of Tendinopathy	Adipose Tissue	Cell Fraction from Adipose Tissue	Treatment	Final Follow-Up and Relative Outcomes
Kim et al. 2017 [55]	Cohort study (Korea)	Rotator cuff disease (MRI)	Lipoaspirated autologous adipose tissue from buttocks, enzymatically digested	ASCs	Intratendinous injections of ASCs (4.46 × 10^6^ cells) during arthroscopic rotator cuff repair	28 months VAS ROM Constant score UCLA score MRI
Jo et al. 2018 [56]	First-in human trial (Korea)	Rotator cuff disease (MRI)	Lipoaspirated autologous abdominal subcutaneous adipose tissue, enzymatically digested	ASCs	Ultrasound-guided intratendinous injection of 3 mL of ASCs at low-(1.0 × 10^7^ cells), mid-(5.0 × 10^7^) and high-dose (1.0 × 10^8^)	6 months SPADI Constant score VAS MRI
Usuelli et al. 2018 [58]	Randomized clinical trial (Italy)	Unilateral or bilateral chronic Achilles tendinopathy (MRI, US)	Lipoaspirated autologous abdominal subcutaneous adipose tissue, processed with Fasti kit System	ASCs in SVF	Ultrasound-guided intratendinous injection of 4 mL of SVF (1.0 × 10^8^)	6 months VAS AOFAS VISA-A SF-36 MRI
Lee et al. 2015 [57]	Open-label study (Korea)	Chronic lateral epicondylosis (US)	Lipoaspirated allogeneic human subcutaneous adipose tissue, enzymatically digested	allo-ASCs	Ultrasound-guided intratendinous injection of in 1 mL allo-ASCs (10^6^ or 10^7^ cells) mixed with fibrin glue	13 months VAS MEPI US
Khoury et al. 2021a [59]	Prospective longitudinal case series exploratory study (Argentina)	Chronic insertional patellar tendinopathy (MRI)	Lipoaspirated autologous adipose tissue from the periumbilical zone, enzymatically digested	ASCs expanded in vitro	Ultrasound-guided intratendinous injection of 6 × 10^6^ ASCs	12 months VAS knee VISA-P Tegner score MRI
Khoury et al. 2021b [60]	Prospective longitudinal case series exploratory study (Argentina)	Chronic lateral elbow tendinopathy (MRI)	Lipoaspirated autologous adipose tissue from the periumbilical zone, enzymatically digested	ASCs expanded in vitro	Ultrasound-guided intratendinous injection of 8 × 10^6^ ASCs	12 months VAS QuickDASH-Compulsory score QuickDASH-Sport score MRI
Freitag et al. 2020 [61]	Case report (Australia)	Elbow tendinopathy (US)	Lipoaspirated autologous abdominal subcutaneous adipose tissue, enzymatically digested	ASCs	Ultrasound-guided intratendinous injection of 1 × 10^9^ ASCs (1 mL) combined with 1 mL autologous PRP in tendon fibril discontinuity/tearing	30 months NPRS PRTEE

Abbreviations: MRI: magnetic resonance imaging; US: ultrasonography; ASCs: adipose-derived mesenchymal stem cells; SVF: stromal vascular fraction; NPS: Numeric Pain Scale; ASES: American Shoulder and Elbow Surgeon; SPADI: safety and shoulder pain and disability index; VAS: visual analog scale; ROM: range of motion; UCLA: University of California, Los Angeles shoulder rating scale; VISA-A: Victorian Institute of Sports Assessment Achilles questionnaire; AOFAS: American Orthopedic Foot and Ankle Society; SF-36: Short Form Health Survey 36; MEPI: modified mayo elbow performance index; NPRS: Numeric Pain Rating Scale; PRTEE: Patient-Rated Tennis Elbow Evaluation; DASH: Disabilities of the Arm, Shoulder and Hand Score; VISA-P: Victorian Institute of Sports Assessment Patellar tendinopathy questionnaire.

### 3.2. Characteristics of Tendinopathic Patients

The description of tendinopathic patients is reported in Table 2, along with the inclusion/exclusion criteria considered in the seven studies.

All the patients enrolled provided written informed consent [55,56,57,58,59,60,61]. Except for the case report describing one male patient [61], the number of patients with tendinopathies described in the remaining six studies ranged from 12 [57] to 35 [55]. The mean age of the patients ranged from 24.4 [57] to 59.2 [55] years; at least 61% of the cases were males in three studies [57,59,60], while female cases accounted for more than 57% in the remaining three studies [55,56,58]. BMI was not reported in three studies [58,60,61], while it ranged from 23.4 to 51.8 in the other records [55,56,57,59]. The most frequent inclusion criteria for these patients were tendon pain and disability for at least 3 months [55,56,57,58,59,60,61], together with failure of conventional treatments [55,56,57,58,59,60,61]. The case report was the only study describing a successful treatment with autologous ASCs although not for tendinopathy but for symptomatic bilateral knee osteoarthritis [61]. Patient compliance with the protocol at follow-up was reported in 57% of the studies [55,57,58,61]. In the remaining three studies, a total of four patients withdrew consent during the study [56,59,60].

### 3.3. Therapeutic Efficacy and Adverse Reactions to ASCs Intratendinous Injections

The significantly modified scores following intratendinous injections of ASCs are summarized in Table 3, along with the adverse reactions reported following ASCs treatment in tendinopathic patients.

Among the seven studies included in the present systematic review, only one record compared a group of tendinopathic patients receiving ASCs intratendinous injections during arthroscopic rotator cuff repair with a different control group of tendinopathic patients treated with arthroscopic rotator cuff repair alone [55]. The other six studies compared scores between patients at baseline prior to intratendinous injections of ASCs at different intermediate time points and at the follow-up [56,57,58,59,60,61]. In only one case, the ASCs in SVF were also compared with the PRP group [58]. All the studies used a single dose of ASCs [55,58,59,60,61], except for two records [56,57]. In particular, the study comparing two ASCs doses showed no significant differences between the two groups [57]. In contrast, the study analyzing three ASCs doses showed the highest dose as the most effective one at all the time points considered [56].

Except for one study reporting only an improvement of the rate of tear recurrence after 28 months [55], the therapeutic efficacy of ASCs in the tendinopathy was evident in almost all studies at the final follow-up, as demonstrated by the improvement of the different scores and by MRI/US [56,57,58,59,60,61]. All the scores also significantly improved at the intermediate time points analyzed [56,57,58,59,60,61], starting from 15 days after ASCs treatment [58] and lasting up to 13 months [57]. Improvement of scores up to 30 months was shown in only one case [61].

Adverse reactions after intratendinous injections of ASCs were reported in three studies [56,59,60]. The most common were pain or subcutaneous hematoma [56,59,60]. However, none of the adverse reactions reported were serious, and all were resolved.

## 4. Discussion

To date, a wide array of treatments have been available for tendon disorders. However, their effectiveness remains ambiguous. Therefore, one of the main controversies of the clinical management of tendinopathies is to determine the real effectiveness of both standard medical treatments and innovative therapeutic approaches in order to assure an improvement in tendon pain and dysfunction.

Currently, some specific treatment strategies are applied for a specific tendon injury, with physical therapy representing one of the most important options, considering its long-term effectiveness [23]. However, since the traditional treatments may not be the most effective options, clinical regenerative therapies for tendinopathies have been developed over the past decade and continue to evolve. The use of ASCs has emerged as a good candidate for tendon healing due to their properties promoting proliferation and tenogenic differentiation [43,44,45]. These properties have been mostly investigated in musculoskeletal disorders [62], while the application in tendon disorders is mainly supported by pre-clinical evidence [45,48,49,50,51,52]. Nevertheless, the efficacy of intratendinous ASCs injections was reported by all seven studies analyzed in the present review. ASCs acted in terms of tendon pain recovery, starting from the second week after treatment and lasting up to 30 months. This was evident not only in the improvement of the VAS score but also by MRI or US evaluations. In this regard, tendon pain recovery induced by ASCs was in line with previous evidence, reporting a rapid and long-lasting pain relief after the application of ASCs in pre-clinical and clinical osteoarthritis, musculoskeletal and neuropathic pain [63,64,65,66]. From the mechanistic point of view, it may be that the therapeutic efficacy of ASCs in tendon pain could be due to ASCs paracrine anti-inflammatory and anti-oxidative activities [67]. Similar hypothesis could be formulated for the effects of ASCs on the tendon structure. It may be that ASCs preserve tendons by reducing the burden of inflammatory damaging mediators released into the injured tissue and promoting local release of new pro-resolutive and trophic factors. A hypothesis of the latter needs confirmation, although the study conducted by Usuelli et al. on human lymphocyte cell culture exposed to ASCs from the autologous SVF fraction paved the way for this. In this study, human lymphocyte cell culture exposed to ASCs showed reduction in the expression levels of the pro-inflammatory interleukin 6 (IL-6) [58].

## 5. Conclusions

The use of ASCs has emerged as a good candidate for tendon healing over the past decade. ASCs isolated from the autologous adipose tissue seem the best candidate, since they lack immune rejection after intratendinous application, while allogeneic ASCs from allogeneic adipose tissue could be immunogenic. By adding paracrine anti-inflammatory and anti-oxidative activities [67] to the well-known pro-proliferation and tenogenic differentiation [43,44,45], ASCs will widen the choice of clinical tools for tendinopathies.

## Figures and Tables

**Table 2:** Characteristics of patients with tendinopathy included in this systematic review.

Reference	Tendinopathy and Treatment	*n* (Baseline)	Age (Years), Sex (%M), BMI (kg/m^2^)	Inclusion Criteria	Exclusion Criteria	*n* (Follow-Up)
Kim et al. 2017 [55]	Rotator cuff disease Intratendinous injection of autologous ASCs during arthroscopic rotator cuff repair	35	59.2, 43%, 26.6	-full-thickness rotator cuff tear diagnosed by a clinical examination, conventional radiography and MRI -shoulder pain and/or functional limitations despite at least 3 months of nonsurgical treatment (anti-inflammatory medication, physical therapy)	- previous surgical treatments - partial or small-sized rotator cuff tears, acromioclavicular arthritis - advanced glenohumeral arthritis	35
Jo et al. 2016 [56]	Rotator cuff disease Ultrasound-guided intratendinous injection of autologous ASCs	20	56.7, 8.3%, 23.4	-19 years of age and older -unilateral shoulder pain of at least 3 months, with failure of conservative treatments -partial-thickness rotator cuff tear confirmed with MRI/US	- shoulder trauma, surgery around shoulder or breast cancer within 6 months - a full-thickness rotator cuff tear or concurrent bilateral shoulder pain - any conditions that could increase the interventional risk - patients pregnant or breast-feeding - anticoagulants − subacromial injection of any drug within 3 months - participation in other clinical trials within 3 months	19
Usuelli et al. 2018 [58]	Unilateral or bilateral chronic tendinopathy of Achilles tendon Ultrasound-guided injection of autologous SVF	21	47.3, 67%, -	-unilateral or bilateral chronic tendinopathy of the Achilles tendon recalcitrant to traditional conservative treatments (NSAIDs, eccentric loading exercises, stretching and biophysical therapy) -symptoms lasting for at least 3 months -age between 18 and 55 -VAS pain >5 at the first visit	- clinical suspicion of other musculoskeletal lesions of the Achilles tendon - platelet count in whole blood <150 × 10^3^/µL - inflammatory disease or other conditions affecting the joints - any immuno-mediated conditions that could increase the interventional risk - use of tendon-detrimental drugs - any previous injective treatment of the target Achilles tendon - patients pregnant or breast-feeding	21
Lee et al. 2015 [57]	Chronic lateral epicondylosis Ultrasound-guided intratendinous injection of allogeneic ASCs mixed with fibrin glue	12	24.4, 42%, 51.8	-lateral elbow pain for at least 6 months -failure of conventional treatments (physical therapy, oral medication, prolotherapy and steroid injection)	- injection therapy within 6 weeks - lateral elbow pain from other musculoskeletal diseases - allergic or hypersensitive reaction to bovine-derived proteins of fibrin glue - any conditions that could increase the interventional risk - patients pregnant or breast-feeding - participation in other clinical trials (involving or not stem cells) within 1 month	12
Khoury et al. 2021a [59]	Chronic insertional patellar tendinopathy Ultrasound-guided intratendinous injection of autologous ASCs	16	34.6, 80%, 23.7	-exercise-related pain located at the patellar tendon insertion for at least 6 months -tenderness to palpation of the tendon substance and thickening and tear of the patellar tendon -failure of exercise-based rehabilitation and different treatment modalities for a minimum of 6 months	- knee or patellar tendon surgeryany inflammatory or prominent degenerative joint condition affecting the knee - contraindication to injection therapy - bleeding diathesis or being on anti-coagulants - local or systemic infection - injections of corticosteroids within 3 months	14
Khoury et al. 2021b [60]	Chronic lateral elbow tendinopathy Ultrasound-guided intratendinous injection of autologous ASCs	19	46.5, 61%, -	-lateral elbow tendinopathy diagnosis -pain and disability for at least 4 months -failure of conventional treatments (eccentric rehabilitation protocol, oral medication, prolotherapy, extracorporeal shock wave therapy, steroid injections, unguided tendon injections with dipropionate and sodium phosphate of betamethasone and PRP)	- lateral elbow pain from other musculoskeletal causes, elbow instability or previous elbow surgery - any other pathology of lateral or collateral ligament tears - upper limb or cervical spine pathology - contraindication to injection therapy - bleeding diathesis or on anti-coagulant - local or systemic infection - injections of corticosteroids within 3 months	18
**Reference**	**Tendinopathy** **and** **Treatment**	***n*** **(Baseline)**	**Age (Years)**, **Sex** **BMI (kg/m^2^)**	**Case Presentation**		***n*** **(Follow-Up)**
Freitag et al. 2020 [61]	Elbow tendinopathy Ultrasound-guided intratendinous injection of autologous ASCs combined with autologous PRP	1	52, M, -	- previous common extensor tendinopathy, treated with physiotherapy and corticosteroid injection - recurrence of pain with increasing pain and debility over the last 3 months - unable to perform simple activities of daily living - previous successful autologous ASCs therapy for symptomatic bilateral knee osteoarthritis - upper limb neural tension testing was negative - large right elbow common extensor origin intrasubstance tear, hypoechoic tendon pattern with loss of fibril continuity, associated florid neovascularization and also fusiform thickening		1

Abbreviations: *n*: number of patients; %M: percentage of males; BMI: body mass index; MRI: magnetic resonance imaging; US: ultrasonography; ASCs: adipose-derived mesenchymal stem cells; SVF: stromal vascular fraction; VAS: visual analog scale; NSAIDs: non-steroidal anti-inflammatory drugs; PRP: platelet-rich plasma.

**Table 3:** Significant score changes and adverse reactions following intratendinous injections of ASCs, reported by the studies included in this systematic review.

Reference	Tendinopathy	Treatment Group (*n*)	Control Group (*n*)	Significant Score at Follow-Up (*p* Value)	Significant Score at Intermediate Time Points (*p* Value)	Score Changes Confirmed by MRI/US	AE Reported (*n* of Patients)
Kim et al. 2017 [55]	Rotator cuff disease	Autologous ASCs in fibrin glue (35)	Arthroscopic rotator cuff repair alone (controls) (35)	**Rate of tear recurrence** Controls: 28.5% ASCs: 14.3% (*p* < 0.001)	-	MRI	-
Jo et al. 2018 [56]	Rotator cuff disease	Autologous ASCs (19) 3 low-dose; 3 mid-dose; 13 high-dose	Baseline (19)	**SPADI** mid baseline: 63.8; 6 months: 12.8 (*p* < 0.05) high baseline: 75.4; 6 months: 17.7 (*p* < 0.001) **Constant score** mid baseline: 60.9; 6 months: 77.1 (*p* < 0.05) high baseline: 55.7; 6 months: 66.9 (*p* < 0.05) **VAS** high baseline: 45.4; 6 months: 25.9 (*p* < 0.001)	high: reduced vs. baseline at month 1 and 3 (both *p* < 0.001) high: increased vs. baseline at month 1 (*p* < 0.001) high: reduced vs. baseline at month 1 and 3 (both *p* < 0.001)	MRI	low: 1 mid: 1 high: 3
Usuelli et al. 2018 [58]	Unilateral or bilateral chronic tendinopathy of Achilles tendon	Autologous SVF containing ASCs (21) autologous PRP (23)	Baseline (21) Baseline (23)	**VAS** SVF and PRP (6 months): reduced vs. baseline (*p* < 0.001) **AOFAS** SVF and PRP (6 months): increased vs. baseline (*p* < 0.001) **VISA-A** SVF and PRP (6 months): increased vs. baseline (*p* < 0.001)	SVF and PRP: reduced vs. baseline at 15 days, month 1–2–4 (all *p* < 0.001) SVF: reduced vs. PRP at 15 days and 1 month (both *p* < 0.05) SVF: increased vs. baseline at 15 days, month 1–2–4 (all *p* < 0.001) PRP: increased at baseline at month 2–4 (both *p* < 0.001) SVF: increased vs. PRP at 15 days (*p* < 0.05) SVF and PRP: increased vs. baseline at month 1–2–4 (all *p* < 0.001) SVF: increased vs. PRP at month 1 (*p* < 0.05)		-
Lee et al. 2015 [57]	Chronic lateral epicondylosis	allogeneic ASCs mixed with fibrin glue (12) 6 low-dose (10^6^) 6 high-dose (10^7^)	baseline (12)	**VAS** baseline: 66.8; all (13 months): 14.8 (*p* < 0.0001) **MEPI** baseline: 64.0; all (13 months): 90.6 (*p* < 0.001)	all reduced vs. baseline at week 6, month 1 and 2 (all *p* < 0.01) all increased vs. baseline at week 6, month 1 and 2 (all *p* < 0.01)	US	-
Khoury et al. 2021a [59]	Chronic insertional patellar tendinopathy	autologous ASCs (14)	baseline (14)	**knee VISA-P** baseline: 43.8; 12 months: 78.7 (*p* < 0.05) **VAS** baseline: 7.4; 12 months: 1.5 (*p* < 0.05) **Tegner score** baseline: 2.3; 12 months: 7.2 (*p* < 0.05)	increased vs. baseline at month 3 and 6 (both *p* < 0.05) reduced vs. baseline at month 3 and 6 (both *p* < 0.05) increased vs. baseline at month 3 and 6 (both *p* < 0.05)	MRI	2
Khoury et al. 2021b [60]	Chronic lateral elbow tendinopathy	autologous ASCs (18)	baseline (18)	**VAS** baseline: 6.28; 12 months: 0.74 (*p* < 0.001) **QuickDASH-Compulsory score** baseline: 51.38; 12 months: 5.76 (*p* < 0.001) **QuickDASH-Sport score** baseline: 56.94; 12 months: 8.68 (*p* < 0.001)	reduced vs. baseline at month 1 and 6 (both *p* < 0.001) reduced vs. baseline at month 1 and 6 (both *p* < 0.001) reduced vs. baseline at month 6 (*p* < 0.001)	MRI	2
Freitag et al. 2020 [61]	Elbow tendinopathy	autologous ASCs combined with autologous PRP (1)	baseline (1)	**NPRS** baseline: 9; 30 months: 0 **PRTEE** baseline: 85; 30 months: 0	baseline: 9; 30 months: 0 baseline: 85; 30 months: 0	US	-

Abbreviations: *n*: number of patients at follow-up; baseline: pre-intratendinous injection; MRI: magnetic resonance imaging; US: ultrasonography; AE: serious adverse events; ASCs: adipose-derived mesenchymal stem cells; SVF: stromal vascular fraction; PRP: platelet-rich plasma; NPS: Numeric Pain Scale; ASES: American Shoulder and Elbow Surgeon; SPADI: safety and shoulder pain and disability index; VAS: visual analog scale; NCI–CTCAE v4.0: National Cancer Institute-Common Terminology Criteria for Adverse Events; VISA-A: Victorian Institute of Sports Assessment Achilles questionnaire; AOFAS: American Orthopedic Foot and Ankle Society; SF-36: Short Form Health Survey 36; MEPI: modified mayo elbow performance index; NPRS: Numeric Pain Rating Scale; PRTEE: Patient-Rated Tennis Elbow Evaluation; DASH: Disabilities of the Arm, Shoulder and Hand Score; VISA-P: Victorian Institute of Sports Assessment Patellar tendinopathy questionnaire.

## Data Availability

The data presented in this study are contained within the article and its supplementary material.

## Supplementary Materials

The following supporting information can be downloaded at: https://www.mdpi.com/article/10.3390/pharmaceutics14061151/s1, Table S1: PRISMA (Preferred Reporting Items for Systematic review and Meta-Analysis) checklist: recommended items to address in a systematic review; Table S2: Checklist for assessing the quality of the study.
