# Supplementary material for: Why Use Adipose-Derived Mesenchymal Stem Cells in Tendinopathic Patients: A Systematic Review
Source: Pharmaceutics. 2022 May 27;14(6):1151. doi: 10.3390/pharmaceutics14061151 (PMC9230128; doi:10.3390/pharmaceutics14061151)
Supplement: Supplementary file 1 [file pharmaceutics-14-01151-s001.zip › pharmaceutics-1665090-supplementary.pdf]

pharmaceutics

Supplemental Materials: Why to use Adipose-derived mesenchymal stem cells in tendinopathic patients: a systematic review

Annalisa Itro [1], Maria Consiglia Trotta [2,*], Roberta Miranda [3], Marco Paoletta [3], Annalisa De Cicco [3], Caterina Claudia Lepre [2], Umberto Tarantino [4], Michele D'Amico [2], Giuseppe Toro [3,^] and Alfredo Schiavone Panni [3,^]

Table S1. PRISMA (Preferred Reporting Items for Systematic review and Meta-Analysis) checklist: recommended items to address in a systematic review (Moher et al., [44])

Section and Topic	Item number	Checklist item	Reported on page
**TITLE**			
Title:	1	Identify the report as a systematic review, meta-analysis, or both	1
**ABSTRACT**			
Structured summary	2	Provide a structured summary including, as applicable: background; objectives; data sources; study eligibility criteria, participants, and interventions; study appraisal and synthesis methods; results; limitations; conclusions and implications of key findings; systematic review registration number	1
**INTRODUCTION**			
Rationale	3	Describe the rationale for the review in the context of what is already known	1-3
Objectives	4	Provide an explicit statement of the question(s) the review will address with reference to participants, interventions, comparators, and outcomes (PICO)	3
**METHODS**			
Protocol and registration	5	Indicate if a review protocol exists, if and where it can be accessed (e.g., Web address), and, if available, provide registration information including registration number	3
Eligibility criteria	6	Specify study characteristics (e.g., PICOS, length of follow-up) and report characteristics (e.g., years considered, language, publication status) used as criteria for eligibility, giving rationale	3
Information sources	7	Describe all information sources (e.g., databases with dates of coverage, contact with study authors to identify additional studies) in the search and date last searched	3
Search strategy	8	Present full electronic search strategy for at least one database, including any limits used, such that it could be repeated	3
Study selection	9	State the process for selecting studies (i.e., screening, eligibility, included in systematic review, and, if applicable, included in the meta-analysis)	3
Data collection process	10	Describe method of data extraction from reports (e.g., piloted forms, independently, in duplicate) and any processes for obtaining and confirming data from investigators	4
Data items	11	List and define all variables for which data were sought (e.g., PICOS, funding sources) and any assumptions and simplifications made	4
Risk of bias in individual studies	12	Describe methods used for assessing risk of bias of individual studies (including specification of whether this was done at the study or outcome level), and how this information is to be used in any data synthesis	4
Summary measures	13	State the principal summary measures (e.g., risk ratio, difference in means)	-
Synthesis of results	14	Describe the methods of handling data and combining results of studies, if done, including measures of consistency (e.g., I2) for each meta-analysis	-

Risk of bias across studies	15	Specify any assessment of risk of bias that may affect the cumulative evidence (e.g., publication bias, selective reporting within studies)	-
Additional analysis	16	Describe methods of additional analyses (e.g., sensitivity or subgroup analyses, meta-regression), if done, indicating which were pre-specified	-
**RESULTS**			
Study selection	17	Give numbers of studies screened, assessed for eligibility, and included in the review, with reasons for exclusions at each stage, ideally with a flow diagram	4-5
Study characteristics	18	For each study, present characteristics for which data were extracted (e.g., study size, PICOS, follow-up period) and provide the citations	5-12
Risk of bias within the studies	19	Present data on risk of bias of each study and, if available, any outcome level assessment (see item 12)	-
Results of individual studies	20	For all outcomes considered (benefits or harms), present, for each study: (a) simple summary data for each intervention group (b) effect estimates and confidence intervals, ideally with a forest plot	5-12
Synthesis of results	21	Present results of each meta-analysis done, including confidence intervals and measures of consistency	-
Risk of bias across studied	22	Presents results of any assessment of risk of bias across studies (see Item 15)	-
Additional analysis	23	Give results of additional analyses, if done (e.g., sensitivity or subgroup analyses, meta-regression [see Item 16])	-
**DISCUSSION**			
Summary of evidence	24	Summarize the main findings including the strength of evidence for each main outcome; consider their relevance to key groups (e.g., healthcare providers, users, and policy makers	12
Limitations	25	Discuss limitations at study and outcome level (e.g., risk of bias), and at review-level (e.g., incomplete retrieval of identified research, reporting bias)	12
Conclusions	26	Provide a general interpretation of the results in the context of other evidence, and implications for future research	12
**FUNDING**			
Funding	27	Describe sources of funding for the systematic review and other support (e.g., supply of data); role of funders for the systematic review	-

Table S2. Checklist for assessing the quality of the study (Kmet et al., [45])

Criteria		Yes (2)	Partial (1)	No (0)	NA
1	Question / objective sufficiently described?				
2	Study design evident and appropriate?				
3	Method of subject/comparison group selection or source of information/input variables described and appropriate?				
4	Subject (and comparison group, if applicable) characteristics sufficiently described?				
5	If interventional and random allocation was possible, was it described?				
6	If interventional and blinding of investigators was possible, was it reported?				
7	If interventional and blinding of subjects was possible, was it reported?				
8	Outcome and (if applicable) exposure measure(s) well defined and robust to measurement / misclassification bias? Means of assessment reported?				
9	Sample size appropriate?				
10	Analytic methods described/justified and appropriate?				
11	Some estimate of variance is reported for the main results?				
12	Controlled for confounding?				
13	Results reported in sufficient detail?				
14	Conclusions supported by the results?				

N/A was marked for items not applicable to a study design (i.e., items 3, 5-11 were not applicable for the case report) and were excluded from the calculation of the summary score. Summary scores of the studies were calculated by scoring the single item when applicable, by summing the scores and dividing by the total possible score (12 for the case report and the cohort-study; 48 for all the studies). The value obtained was expressed as percentage. The minimum quality score for inclusion was 65%.
